# Circadian rest–activity rhythm disruption and ventricular arrhythmias in implantable cardioverter-defibrillator patients: a prospective wearable study

**DOI:** 10.1093/europace/euag216

**Published:** 2026-08-18

**Authors:** Femke D Raijmakers, Diana My Frodi, Joss Langford, Hanno L Tan, Tariq O Andersen, Samuel Ruipérez-Campillo, Peter Karl Jacobsen, Niels Risum, Søren Zöga Diederichsen, Reinoud E Knops, Jesper Hastrup Svendsen, Maarten Z H Kolk, Fleur V Y Tjong

**Affiliations:** Department of Clinical and Experimental Cardiology, Amsterdam UMC, Heart Center, Meibergdreef 9, 1105AZ Amsterdam, The Netherlands; Heart Failure & Arrhythmias, Amsterdam Cardiovascular Sciences, Meibergdreef 9, 1105AZ Amsterdam, The Netherlands; Department of Cardiology, Copenhagen University Hospital – Rigshospitalet, Inge Lehmanns Vej 7, DK-2100 Copenhagen, Denmark; Department of Public Health and Sport Sciences, University of Exeter, Exeter, UK; ActivInsights Ltd, Kimbolton, UK; Department of Clinical and Experimental Cardiology, Amsterdam UMC, Heart Center, Meibergdreef 9, 1105AZ Amsterdam, The Netherlands; Department of Computer Science, University of Copenhagen, Copenhagen, Denmark; Department of Computer Science, ETH Zurich, Zurich, Switzerland; AI Center, ETH Zurich, Zurich, Switzerland; Department of Cardiology, Copenhagen University Hospital – Rigshospitalet, Inge Lehmanns Vej 7, DK-2100 Copenhagen, Denmark; Department of Cardiology, Copenhagen University Hospital – Rigshospitalet, Inge Lehmanns Vej 7, DK-2100 Copenhagen, Denmark; Department of Cardiology, Copenhagen University Hospital – Rigshospitalet, Inge Lehmanns Vej 7, DK-2100 Copenhagen, Denmark; Department of Clinical and Experimental Cardiology, Amsterdam UMC, Heart Center, Meibergdreef 9, 1105AZ Amsterdam, The Netherlands; Heart Failure & Arrhythmias, Amsterdam Cardiovascular Sciences, Meibergdreef 9, 1105AZ Amsterdam, The Netherlands; Department of Cardiology, Copenhagen University Hospital – Rigshospitalet, Inge Lehmanns Vej 7, DK-2100 Copenhagen, Denmark; Department of Clinical Medicine, Faculty of Health and Medical Sciences, University of Copenhagen, Copenhagen, Denmark; Department of Clinical and Experimental Cardiology, Amsterdam UMC, Heart Center, Meibergdreef 9, 1105AZ Amsterdam, The Netherlands; Heart Failure & Arrhythmias, Amsterdam Cardiovascular Sciences, Meibergdreef 9, 1105AZ Amsterdam, The Netherlands; Department of Clinical and Experimental Cardiology, Amsterdam UMC, Heart Center, Meibergdreef 9, 1105AZ Amsterdam, The Netherlands; Heart Failure & Arrhythmias, Amsterdam Cardiovascular Sciences, Meibergdreef 9, 1105AZ Amsterdam, The Netherlands; Department of Medicine, Cardiovascular Institute, Stanford University School of Medicine, Stanford, CA, USA

**Keywords:** Implantable cardioverter defibrillators, Circadian rest-activity rhythm, Risk stratification, Remote monitoring, Accelerometer

## Introduction

In patients with an implantable cardioverter-defibrillator (ICD), prediction of future ventricular fibrillation (VF) or sustained ventricular tachycardia (VT) events that require device therapy [shock or antitachycardia pacing (ATP)] remains challenging.^1,2^ Disruption of circadian rhythms, through intrinsic factors (e.g. disease progression) and extrinsic factors (e.g. shift work or irregular sleep) has been linked to adverse cardiovascular outcomes such as atrial fibrillation (AF), ischaemic heart disease, and exacerbation of dilated cardiomyopathy.^3,4^ However, its predictive value for malignant ventricular arrhythmias in ICD patients remains unclear. A prior substudy of the SafeHeart cohort demonstrated that lower physical activity and disrupted sleep were associated with appropriate ICD therapy.^5^ That analysis focused on total activity volume and sleep instead of the temporal organization of the rest–activity cycle. We investigated whether non-parametric circadian rest–activity rhythm (CRAR) metrics, capturing the pattern and regularity of the 24 h activity rhythm, are associated with appropriate ICD therapy and whether dynamic changes in CRAR precede arrhythmic events.

## Methods

### Data collection

This substudy of the prospective, multicentre SafeHeart study was performed at Amsterdam UMC, Netherlands and Rigshospitalet, Denmark.^6^ Patients with an ICD or cardiac resynchronization therapy defibrillator were enrolled and wore a wrist-based triaxial accelerometer (GENEActiv) for 6 months. Days with more than 1 h of non-wear were excluded. The primary outcome was appropriate ICD therapy (shock and/or ATP) for VF or sustained VT during 12 months of follow-up.

### Circadian rest–activity rhythm

Seven CRAR metrics were derived from the first seven consecutive monitoring days. Two metrics were central to this analysis: CRAR relative amplitude and intradaily variability. CRAR relative amplitude quantifies the contrast between the most and least active periods of the 24 h activity rhythm, computed as $\frac{M10\;-\;L5}{M10\;+\;L5}$, where M10 and L5 are mean activity in the most active 10 h and least active 5 h, respectively. A high CRAR relative amplitude indicates active days and restful nights, while a low amplitude indicates less activity during the day, more restlessness at night, or both. Intradaily variability captures fragmentation of the rest–activity rhythm, with high values indicating frequent switches between rest and activity rather than sustained periods.

### Statistical analysis

Cox regression assessed associations between baseline CRAR and outcomes, adjusted for age, sex, ICD indication, AF, and heart failure with reduced ejection fraction (HFrEF). Temporal trends in CRAR during the 14 days preceding appropriate therapy were examined using mixed-effects models in the 31 participants with accelerometer data available before shock/ATP.

## Results

Of 274 participants (mean age 63 ± 10 years; 18% female), 47 (17%) received appropriate ICD therapy during follow-up (6 shocks, 37 ATP, and 4 both). ICD therapy recipients more often had HFrEF (70.2% vs. 50.7%, *P* = 0.016) and used β-blockers (93.6% vs. 77.5%, *P* = 0.009), with no difference in AF prevalence (36.2% vs. 35.7%, *P* = 1.000).

**Figure 1:**
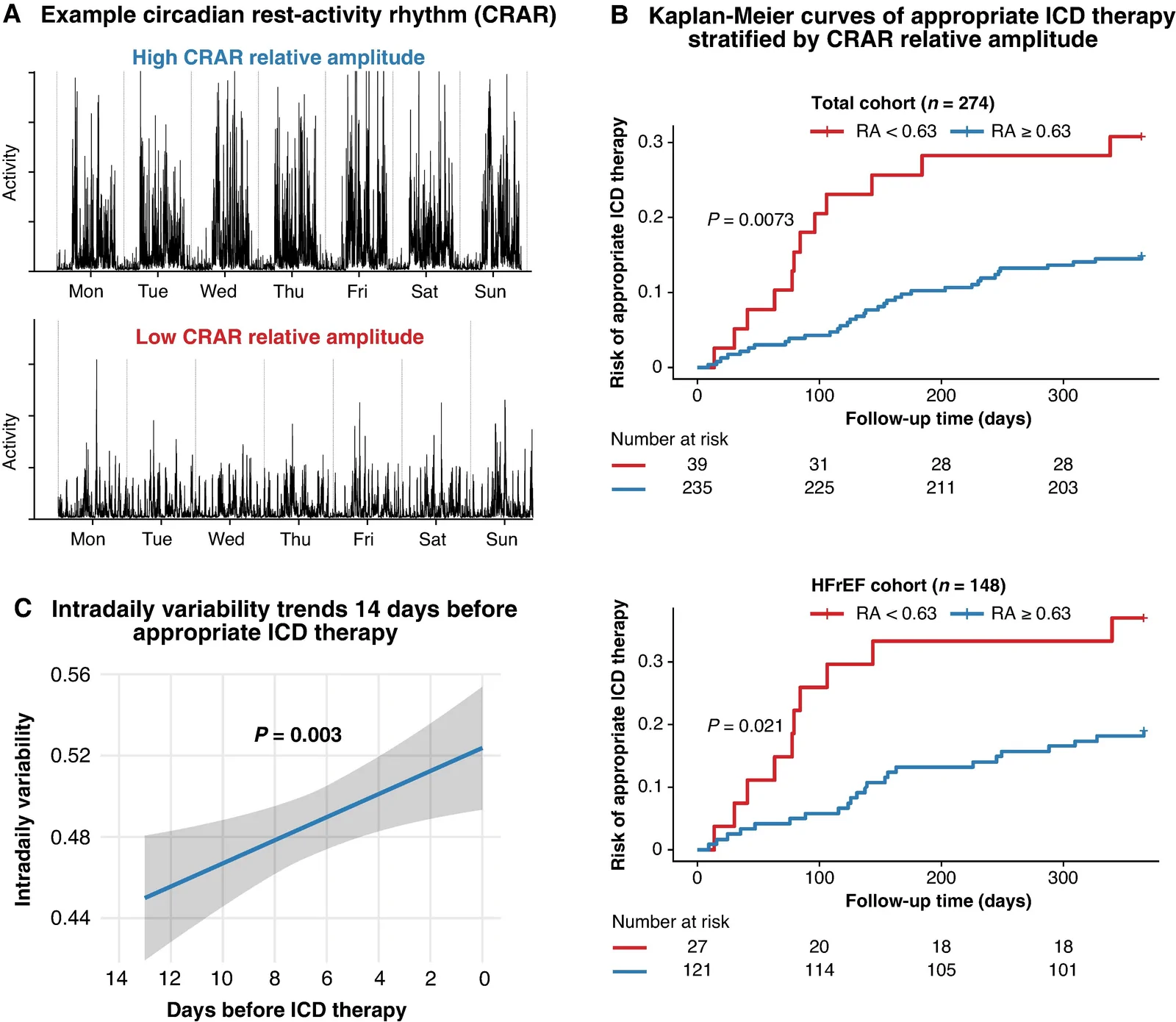
Overview of circadian rest-activity rhythm (CRAR) and main study findings. (*A*) Examples of one-week accelerometer data with a high (top) and low (bottom) CRAR relative amplitude. (*B*) Kaplan-Meier curves showing a significantly higher risk of appropriate ICD therapy (anti-tachycardia pacing and/or shock) in patients with a CRAR relative amplitude below 0.63, in both the overall cohort and the heart failure with reduced ejection fraction (HFrEF) subgroup. (*C*) Significant upward trend in CRAR intradaily variability among 31 ICD patients during the 14 days preceding appropriate ICD therapy.

CRAR relative amplitude was lower in participants receiving ICD therapy (0.69 vs. 0.73, *P* = 0.010). In adjusted Cox regression, each 0.1-unit decrease in amplitude was associated with a 30% higher risk of appropriate ICD therapy (HR 0.77, 95% CI 0.62–0.96). A data-driven cut-off of CRAR relative amplitude <0.63 identified a high-risk subgroup (*n* = 39) with a 4.8-fold higher risk of appropriate therapy at 100 days (*P* = 0.007) using Kaplan–Meier analysis (*Figure 1B*). This group also carried a significantly higher comorbidity burden, including older age, diabetes, hypertension, and AF.

Intradaily variability was elevated at baseline in patients with HFrEF and increased significantly in the 2 weeks preceding ICD therapy (+0.0046/day, *P* = 0.003, *Figure 1C*). This effect was significant over 7 and 14 days but not over 21 days, suggesting short-term physiological deterioration.

## Discussion

This prospective, multicentre SafeHeart substudy explores the relation between CRAR disruption and malignant ventricular arrhythmias in ICD patients. Findings showed that a lower CRAR relative amplitude was independently associated with appropriate ICD therapy, whereas intradaily variability increased 2 weeks preceding VT/VF.

This is the first study to investigate CRAR in relation to VT/VF risk using long-term accelerometer recordings. Prior studies showed that low daily activity and disturbed sleep are associated with appropriate ICD therapy.^5^ This work extends this by showing that the pattern of the rest–activity cycle rather than absolute activity levels, is associated with arrhythmic risk.

Participants with lower CRAR relative amplitude also carried a higher comorbidity burden. However, these comorbidities were not more prevalent among patients who received appropriate ICD therapy, suggesting that CRAR relative amplitude captures physiological vulnerability beyond traditional risk factors. Whether a low CRAR relative amplitude increases arrhythmic vulnerability or indicates general health decline, or both, cannot be determined from this observational design.

Intradaily variability was higher at baseline in patients with HFrEF and increased further in the 2 weeks preceding ICD therapy. This pattern, more frequent transitions between rest and activity mirrors a clinically recognizable pattern in decompensating heart failure patients with increasing fatigue during the day and disturbed sleep at night (e.g. orthopnoea, nocturia), producing a chaotic rather than structured activity profile.^7^ The effect was significant over 7 and 14 days but not 21 days, consistent with acute physiological decline rather than a long-term process.

From a monitoring perspective, CRAR relative amplitude represents a static marker of circadian organization, suitable for periodic risk assessment. Intradaily variability is a dynamic marker requiring continuous monitoring in high-risk patients. A relative amplitude below 0.63 identified a subgroup with an almost five-fold higher risk of appropriate therapy within 100 days. If confirmed in a larger cohort, integration of CRAR monitoring into ICD follow-up could provide clinically actionable information. Circadian disruption may also represent a modifiable target; interventions such as structured activity, sleep optimization, or light therapy warrant further investigation.

## Conclusion

Lower relative amplitude of the CRAR, reflecting a blunted contrast between active days and restful nights, is independently associated with a higher risk of appropriate ICD therapy. Progressive fragmentation of the activity pattern is detectable in the 2 weeks preceding arrhythmic events. Prospective validation in larger, more diverse cohorts is needed, but these findings suggest that wearable-derived CRAR metrics may contribute to both static risk stratification and dynamic early warning in ICD patients.

## Data Availability

The data underlying this article will be shared on reasonable request to the corresponding author.
